# Site-Specific Keloid Fibroblasts Alter the Behaviour of Normal Skin and Normal Scar Fibroblasts through Paracrine Signalling

**DOI:** 10.1371/journal.pone.0075600

**Published:** 2013-12-09

**Authors:** Kevin J. Ashcroft, Farhatullah Syed, Ardeshir Bayat

**Affiliations:** 1 Plastic & Reconstructive Surgery Research, Manchester Institute of Biotechnology, University of Manchester, United Kingdom; 2 Institute of Inflammation & Repair, University of Manchester, Manchester, United Kingdom; 3 Department of Plastic and Reconstructive Surgery, University Hospital South Manchester Foundation Trust, Wythenshawe Hospital, Manchester, United Kingdom; 4 The University of Manchester, Manchester Academic Health Science Centre, University Hospital South Manchester Foundation Trust, Wythenshawe Hospital, Manchester, United Kingdom; National Center for Scientific Research Demokritos, Greece

## Abstract

Keloid disease (KD) is an abnormal cutaneous fibroproliferative disorder of unknown aetiopathogenesis. Keloid fibroblasts (KF) are implicated as mediators of elevated extracellular matrix deposition. Aberrant secretory behaviour by KF relative to normal skin fibroblasts (NF) may influence the disease state. To date, no previous reports exist on the ability of site-specific KF to induce fibrotic-like phenotypic changes in NF or normal scar fibroblasts (NS) by paracrine mechanisms. Therefore, the aim of this study was to investigate the influence of conditioned media from site-specific KF on the cellular and molecular behaviour of both NF and NS enabled by paracrine mechanisms. Conditioned media was collected from cultured primary fibroblasts during a proliferative log phase of growth including: NF, NS, peri-lesional keloid fibroblasts (PKF) and intra-lesional keloid fibroblasts (IKF). Conditioned media was used to grow NF, NS, PKF and IKF cells over 240 hrs. Cellular behavior was monitored through real time cell analysis (RTCA), proliferation rates and migration in a scratch wound assay. Fibrosis-associated marker expression was determined at both protein and gene level. PKF conditioned media treatment of both NF and NS elicited enhanced cell proliferation, spreading and viability as measured in real time over 240 hrs versus control conditioned media. Following PKF and IKF media treatments up to 240 hrs, both NF and NS showed significantly elevated proliferation rates (p<0.03) and migration in a scratch wound assay (p<0.04). Concomitant up-regulation of collagen I, fibronectin, α-SMA, PAI-1, TGF-β and CTGF (p<0.03) protein expression were also observed. Corresponding qRT-PCR analysis supported these findings (P<0.03). In all cases, conditioned media from growing marginal PKF elicited the strongest effects. In conclusion, primary NF and NS cells treated with PKF or IKF conditioned media exhibit enhanced expression of fibrosis-associated molecular markers and increased cellular activity as a result of keloid fibroblast-derived paracrine factors.

## Introduction

Keloid disease (KD) is a complex fibroproliferative disorder of the skin characterised by formation of raised dermal lesions following an abnormal wound healing response [1]. The aetiopathogenesis of KD has yet to be elucidated, although both environmental and genetic risk factors have been implicated [2]. Histopathologically, KD scars are characterised by rich vasculature [3], [4], a thickened epidermis and a high mesenchymal cell density [5]. These features are accompanied by thick compact hyalinised collagen fibres [6] forming whirl-like nodular structures in the reticular dermis [7], [8]. This irregular collagen distribution forms the basis for the dense ECM meshwork within the KD lesion [5] and is distinct from normal skin, in which collagen bundles appear parallel to the epidermis [9]. The main inductive cells for KD are thought to be keloid fibroblasts (KF), which initially show a marked infiltration in lesion tissue and subsequently mediate elevated pro-collagen I/III expression [10]. However, the exact mechanisms by which KF potentiate keloid scar formation and invasion, remain to be fully characterised.

It is possible that KF contribute to disease pathogenesis by possessing genetic or epigenetic variations [11], potentiating abnormal secretory and/or responsive behaviour to cues in the lesional micro-environment [12], . Indeed, elevated cytokine production has been detected in KF conditioned media compared to normal dermal fibroblasts (NF) from non co-culture conditions [14]. Aberrant KF behaviour may also be augmented through KD mesenchymal-epithelial interactions, inducing changes such as increased collagen production [15], connective tissue growth factor (CTGF) expression [16] and contraction of fibroblast-populated collagen gels [17]. Paracrine support from normal connective tissue fibroblasts during healthy cutaneous wound healing is thought saliant for efficient re-epithelialization of deeper dermal defects, where the supporting growth factor incentive is absent [18]. In these healthy individuals, subsequent re-epithelialisation is deemed necessary to counteract excessive/fibrotic scar formation [19]. Any changes to the overall complement of growth factors, chemokines or cytokines in the wound micro-environment, either from KF inherently or as a result of epithelial-mesenchymal influence, may thus contribute towards aberrant physiological repair processes as occur in KD.

Transforming growth factor (TGF)-β is a key cytokine involved in the initiation and termination of tissue repair, whose sustained production underlies development of tissue fibrosis and whose expression is thought to be up-regulated in KF [20], [21]. The chemotactic activity of vascular endothelial cells is strongly induced by KF conditioned media, resulting from endogenous TGFβ-mediated up-regulation of fibroblast vascular endothelial growth factor (VEGF) [20]. Additionally, exogenous TGFβ may stimulate significantly higher collagen I expression in KF versus normal skin fibroblasts [22], [23] and work synergistically with insulin-like growth factor (IGF)-1 to induce markedly higher expression of collagen I, fibronectin and plasminogen activator inhibitor (PAI)-1 [23]. These results indicate that KF, in addition to producing more TGFβ, may respond inappropriately to its production through autocrine and/or paracrine mechanisms and that ultimately, multiple secreted factors may influence a fibrotic phenotype.

The locality of KF within the lesion is also known to be intricately linked to the extent of collagen I/III synthesis, with KF cultured from the peri-lesional (growing margin) area producing more collagen I and III *in vitro* compared to those cultured from intra-lesional (reticular dermis of scar) or extra-lesional (surrounding normal skin) sites [1]. Also, observations indicate the fibrotic marker connective tissue growth factor (CTGF) is more concentrated in KF at the expanding invasive border of the KD scar [24]. Indeed, array profiling of KF have also shown differential region-specific gene expression patterns [25], [26]. These results collectively suggest that the site of the scar from which KF are specifically isolated may dictate or influence a certain type of cellular behaviour.

We hereby hypothesise that aberrant or enhanced secretory activity may occur in site-specific KF compared with healthy NF and normal scar fibroblasts (NS). Furthermore, these paracrine effects may influence expression of fibrosis-associated markers in both NF and NS cells. The objective of our study was to therefore investigate the influence of conditioned media from site-specific KF, i.e. from the invasive margin (peri-lesional keloid fibroblasts) or central reticular dermis of the scar (intra-lesional keloid fibroblasts), on the cellular and molecular behaviour of both NF and NS as mediated through paracrine mechanisms.

## Materials and Methods

### Patients and tissue samples

**Figure 1:**
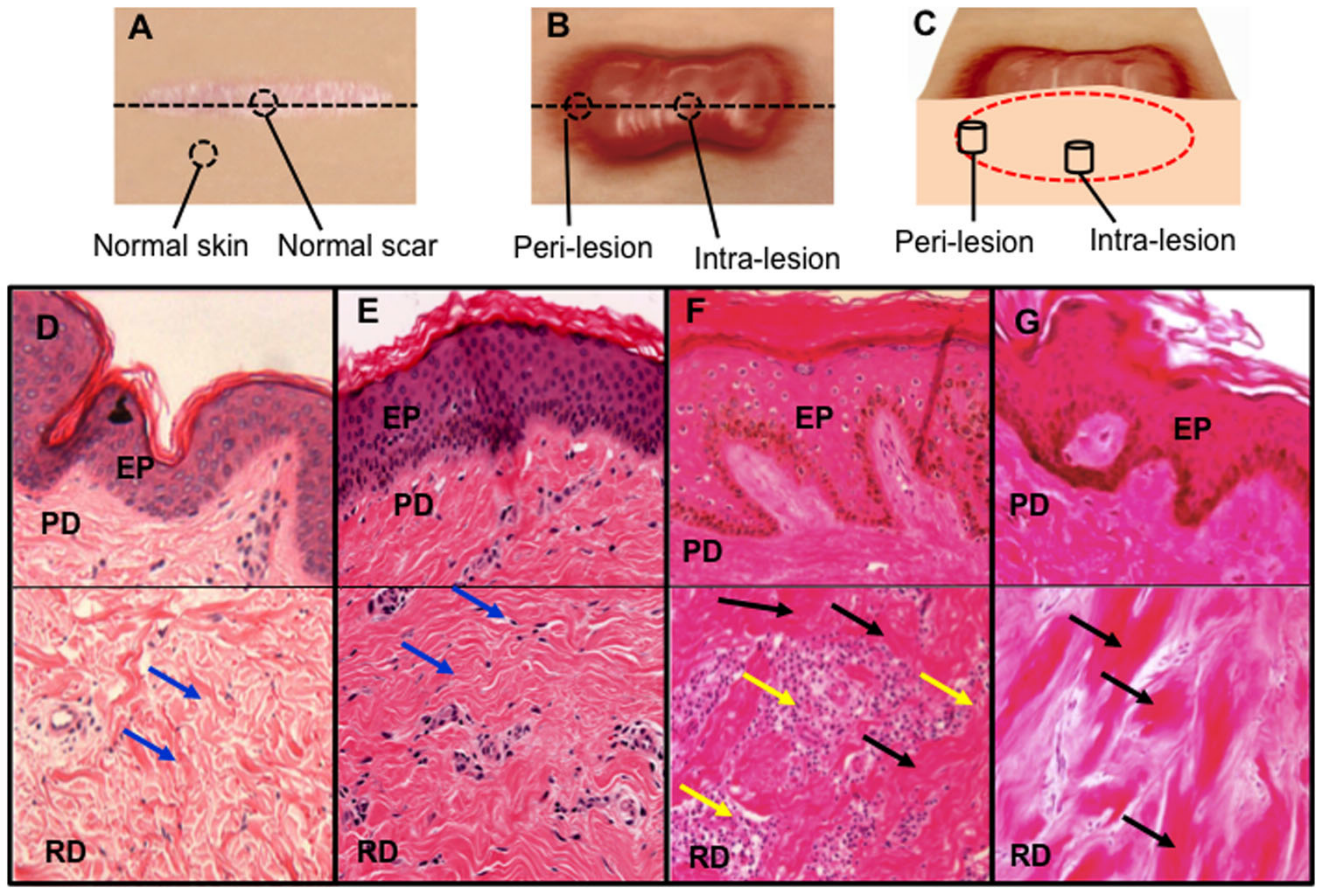
Dermal biopsy locations from healthy controls and keloid patients with corresponding histology. **A.** Transverse view of biopsy locations from normal dermal scar tissue and adjacent normal dermal (non-wounded) skin from which *in vitro* primary cell cultures were subsequently established. **B.** Transverse view of marginal peri-lesional and reticular dermal intra-lesional biopsy sites from the keloid scar. **C.** Cross-section of keloid scar indicating depth of peri-lesional and intra-lesion biopsies. **D.** Representative H&E staining of tissue section from normal skin indicating organised wavy deposition of collagen (arrows). **E.** Representative H&E staining of tissue section from a normal scar. **F.** Representative H&E staining of a peri-lesional keloid tissue section indicating a thickened EP with increased cell infiltration (yellow arrow) and deposition of hyalinised collagen bundles in the RD (black arrow). **G.** Representative H&E staining of an intra-lesional keloid tissue section indicating thick compact hyalinised collagen bundle deposition in the RD (black arrow). EP = Epidermis, PD = Papillary dermis, RD = Reticular dermis. All the H&E micrographs (D–G) were taken at 200 magnifications.

Keloid tissues were harvested at the time of surgery from patients confirmed to have clinical and pathological evidence of keloid, were ethically consented (ethical approval was obtained from NHS Ethical Committee, Manchester, UK). All cases recruited gave full verbal and written consent to take part in the study (ethical reference number 11/NW/0638). Following surgical excision, KD biopsy tissue was sectioned into two specific lesional sites: Peri-lesional from the growing margin of the scar adjoining the normal non-affected skin (n = 5) and intra-lesional from the reticular dermal centre of the scar (n = 5). Normal skin biopsies (n = 4) were obtained adjacent to a normal scar during scar revision surgery. Normal scar biopsies (n = 4) were defined as a flat scar in an individual with no previous personal or family history of hypertrophic or keloid scarring. Both normal skin and normal scars were used as controls in our study (**Table 1** **and** **Figure 1A**). Patient recruitment was determined using stringent diagnostic criteria with each KD scar confirmed histologically (**Figure 1B**), limiting any confounding effects of disease heterogeneity or misdiagnosis. KD scars were defined as tumour-like lesions that extended beyond the margin of the initial underlying wound, continued to grow over time without spontaneous regression, commonly recurred following excision and were present for a minimum of one year. Complete clinical records including detailed data on scar history were obtained (including cause, symptoms, past medical history, duration of scar and previous scar treatment).

**Table 1:** Demographic data for keloid patients and control subjects.

Subject	Biopsy	Patient age	Sex	Age of scar	Location of surgery	Ethnicity
**Keloid 1**	Per-lesional	31	M	2 years	Scalp	BC
**Keloid 2**	Per-lesional	42	M	24 years	Sternum	BC
**Keloid 3**	Per-lesional	36	F	28 years	Earlobe	BC
**Keloid 4**	Per-lesional	74	M	11 years	Sternum	A
**Keloid 5**	Per-lesional	34	M	5 years	Sternum	BC
**Keloid 1**	Intra-lesional	31	M	2 years	Scalp	BC
**Keloid 2**	Intra-lesional	42	M	24 years	Sternum	BC
**Keloid 3**	Intra-lesional	36	F	28 years	Earlobe	BC
**Keloid 4**	Intra-lesional	74	M	11 years	Sternum	A
**Keloid 5**	Intra-lesional	34	M	5 years	Sternum	BC
**Normal skin 1**	Cross section	31	M	2 years	Chest	W
**Normal skin 2**	Cross section	39	F	1 year	Shoulder	W
**Normal skin 3**	Cross section	54	F	7 years	Cheek	W
**Normal skin 4**	Cross section	41	F	36 years	Shoulder	W
**Normal scar 1**	Cross section	41	F	36 years	Shoulder	W
**Normal scar 2**	Cross section	36	F	2 years	Eyelid	W
**Normal scar 3**	Cross section	32	F	5 years	Sternum	W
**Normal scar 4**	Cross section	20	F	3 years	Upper arm	W

### Establishment of primary fibroblast cultures from keloid scar, normal scar and normal skin

Cultures were established from tissue specimens processed within 6 hrs post-surgical excision as described previously [3], [4]. Briefly, biopsies were washed 3 times in 1× phosphate buffered saline (PBS) (PAA, Germany) and incubated in Dispase II, 10 mg/ml (Roche, UK) for 3 hrs at 37°C/5%CO_2_ humidified atmosphere. The epidermis and subdermal fat were removed and the remaining dermis dissected and incubated in 0.5 mg/ml collagenase I (Roche, UK) for 4 hrs at 37°C/5%CO_2_. Cells were then centrifuged, pelleted and re-suspended in complete DMEM (containing 2 mmol/L L-glutamine (PAA, Germany), 100 U/ml penicillin and 100 U/ml streptomycin (PAA, Germany), 1% non-essential amino acids (Sigma-Aldrich, UK) and 250 µg/ml amphotericin-B (Sigma-Aldrich, UK)) with 10% heat-inactivated foetal calf serum (FCS) (Sigma-Aldrich, UK). The entire re-suspended cell pellet, with residual tissue explants, was transferred into T25 CellBind flasks (Nunc, Life Technologies Ltd., Germany) and monolayer cultures subsequently established at 37°C/5%CO_2_.

### Haematoxylin and eosin staining

Keloid site-specific scar samples were fixed in formaldehyde, embedded in paraffin blocks and sectioned to 5 µm thicknesses. The sections were then stained with haematoxylin and eosin (H&E) (Surgipath, Peterborough, U.K.) for histological evaluation using standardised protocols established in the lab as described previously [27], [28]. Micrographs were taken at 200 magnification using Olympus microscopy (BX51, Olympus, UK).

### Collection of conditioned media from primary fibroblast cultures

**Figure 2:**
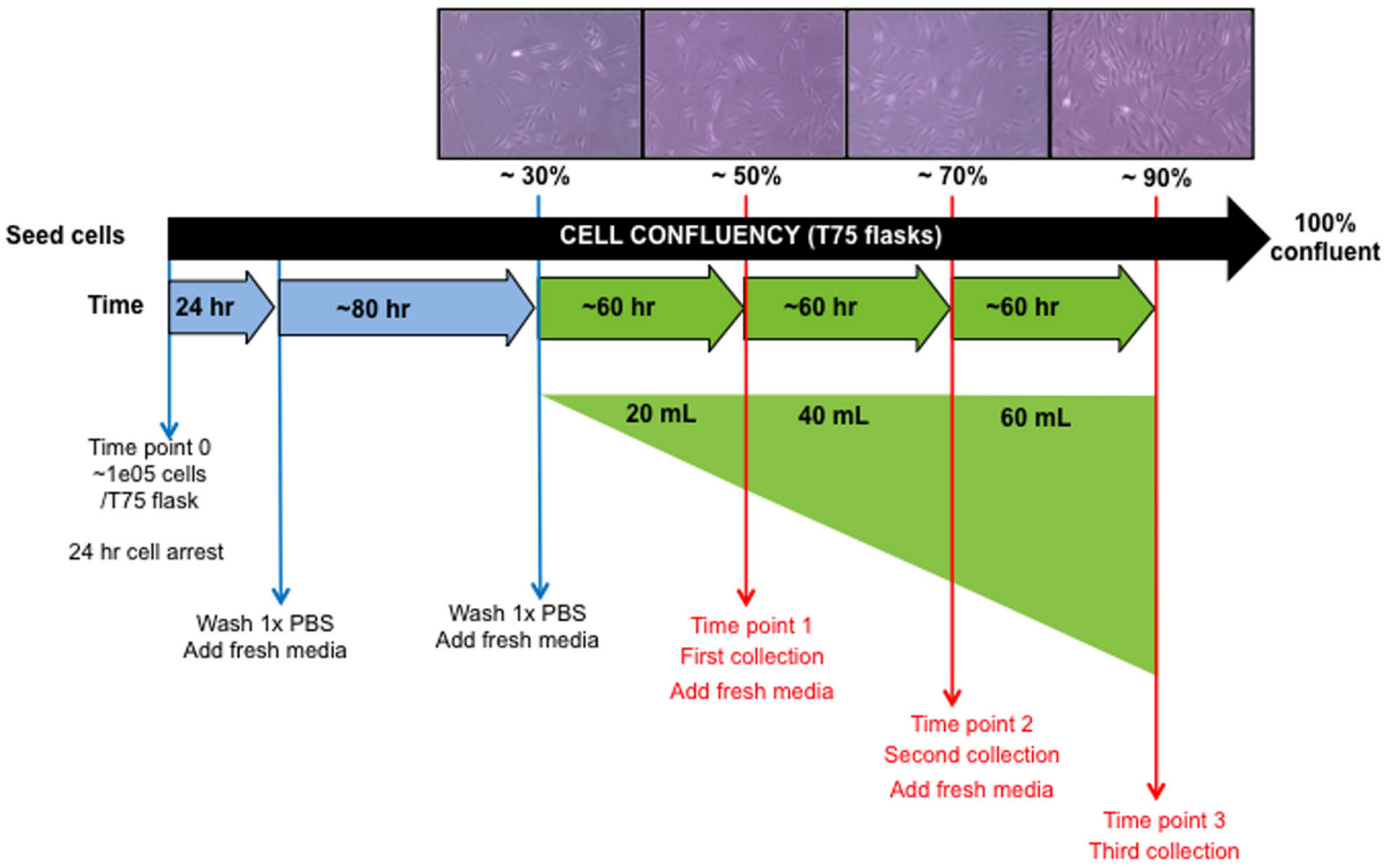
Schematic timeline for collection of primary culture conditioned media. Conditioned media was collected every ∼60 hrs when PKF (n = 5), IKF (n = 5), NF (n = 4) and NS (n = 4) were in proliferative log phase of growth. For each individual primary cell culture, conditioned media was collected at ∼50%, 70% and 90% confluency states and subsequently amalgamated prior to treatment of cells. PKF = peri-lesional keloid fibroblasts, IKF = intra-lesional keloid fibroblasts, NF = normal skin fibroblasts, NS = normal dermal scar fibroblasts.

Once confluent peri-lesional keloid fibroblasts (PKF), intra-lesional keloid fibroblasts (IKF), normal dermal fibroblasts (NF) and normal scar tissue fibroblasts (NS) were established as monolayer cultures (n = 16), passaging of cells into T75 flasks (Nunc, Life Technologies Ltd., Germany) facilitated collection of conditioned media every ∼60 hrs when each cell type was in a proliferative log phase of growth. Collection was started at 30% confluency, ∼1.0e4 cells, when cells were actively dividing and having formed lamellipodial and filopodial projections. After each media collection, cells were washed in 1×PBS and 20 mL new complete DMEM was placed onto cells. The conditioned media was then collected corresponding to approx. 50, 70 and 90% confluency levels (**Figure 2**). Collection periods were ∼60 hrs apart depending on cell type. PKF and IKF cells were not used beyond passage 3 in order to retain KD-like properties [1]. Conditioned media was stored at −80°C until required, then thawed overnight (4°C) and subsequently filtered through a 0.2 µm membrane (AppletonWoods, UK). Each sample collection time-point was amalgamated prior to use (e.g. patient 1 PKF media representing 30–50%, 50–70% and 70–90% confluency states were combined).

### Treatment of target primary fibroblasts with conditioned media

**Figure 3:**
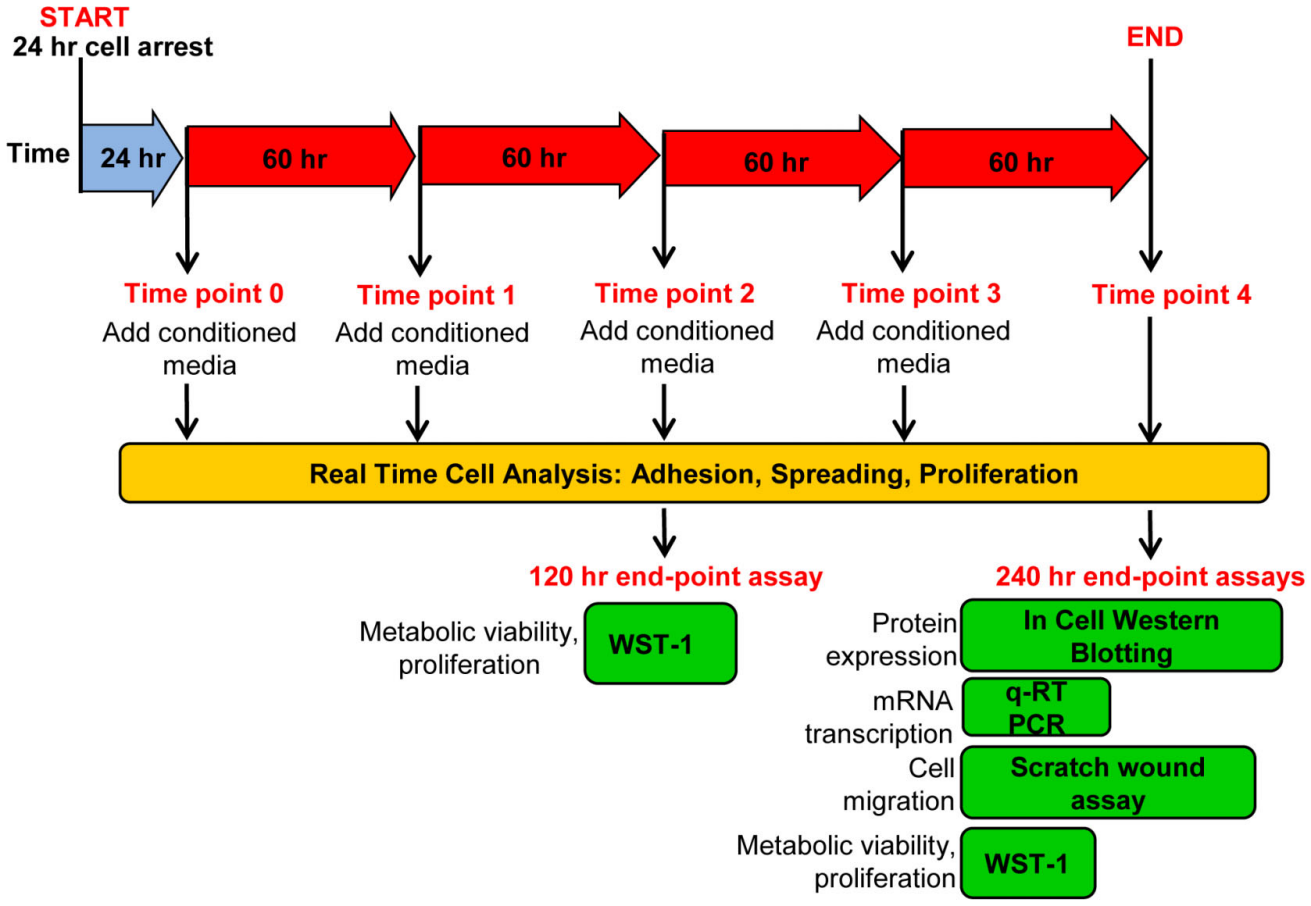
Schematic timeline for treatment of primary culture cells with conditioned media. NF (n = 4), NS (n = 4), PKF (n = 5), and IKF (n = 5) were independently treated with all the different conditioned media types (n = 16) following 24 hrs cell synchronisation. Conditioned media was continually replaced every 60 hrs up to a final treatment period of 240 hrs. RTCA on a micro-sensory array was undertaken for the complete 240 hrs whereas end-point assays were conducted at either 120 hrs or 240 hrs. PKF = peri-lesional keloid fibroblasts, IKF = intra-lesional keloid fibroblasts, NF = normal dermal fibroblasts, NS = normal dermal scar fibroblasts, RTCA = Real time cell analysis.

PKF, IKF, NF and NS fibroblasts were grown in monolayers to confluency in T75 flasks. Cells were trypsinised (PAA, UK), re-suspended in 10% FCS complete DMEM and counted using the Scepter 2.0 automated counter (Millipore, UK). 1.0e4 cells/well were seeded into 96-well plates (Corning, UK) or 3.0×10^4^ cells/well in 24-well plates (Corning, UK) in triplicates and allowed to attach to the well surface. Cell-synchronisation was performed by starving cells in complete 0.2% serum DMEM for 24 hrs. Cells were washed once with 1× PBS and then conditioned media gently added (150 µL/well in 96-well plate or 500 ul/well 24-well plate). Plates were incubated at 37°C/5% CO_2_. All media types were replaced with corresponding fresh conditioned media after consecutive 60 hrs periods, following a single 1× PBS wash. End-point assays were performed on cells after 120 hrs or 240 hrs of replenishing conditioned media treatments (**Figure 3**).

### Label free Real-Time Cell Analysis: RTCA (cell attachment, proliferation, spreading)

The RTCA system monitors change in electrode impedance caused by the interaction between testing cells and electrodes. Electronic impedance is primarily determined by the ion environment both at the electrode/solution interface and in the bulk solution. When an electric field is applied, ions undergo field directed movement leading to frequency dependent impedance dispersion. The presence of cells affects the local ionic environment leading to an increase in the electrode impedance [29]. Cell Index (CI) is used to represent cell status based on the measured frequency-dependent electrical impedance. CI maybe used as a global guide to cellular behaviour including attachment, proliferation and cell spreading. Initially, 50 µL complete DMEM was added to each well of a 96-well plate microelectronic sensory array (MESA). The plate was pre-incubated at 37°C/5%CO_2_ to achieve temperature equilibration prior to cell seeding. 1.0×10^4^ cells were added to the 96-well MESA plate depending on the experimental plate design, with three cell types (treated with different conditioned media) assessed in each plate. An xCELLigence instrument (Roche, UK), housed within a 37°C/5%CO_2_ incubator, was used to assess CI values every 15 min over ∼7–8 hrs. After initial cell attachment to the well surface, the plate was washed once with 1× PBS and 150 µL conditioned media added to the appropriate wells in triplicate and CI further assessed every 15 min. After consecutive 60 hrs periods, corresponding fresh conditioned media was added to the cells following a single 1× PBS wash. CI was measured on a continual basis (every 15 min) up to 240 hrs. The protocol is well established for primary fibroblasts [3], [4], [30], [31].

### WST-1 (cellular proliferation/viability)

WST-1 proliferation assay (Roche diagnostics, UK) was performed according to the manufactures instructions, following different conditioned media treatments on our panel of primary fibroblast cultures after 240 hrs treatments. Briefly, cells were washed in 1× PBS and 90 µl complete 10% serum DMEM (non-conditioned) was added along with 10 µl WST-1 reagent. Plates were agitated gently and then incubated at 37°C/5% CO_2_ for 4 hrs. During this incubation period any viable cells converted the stable tetrazolium salt (WST-1) to a water soluble formazan dye, largely dependent on the bio-reduction of glycolysis produced NAD(P)H, thus acting as a direct measure of cell number/viability. Absorbance was measured at 450 nm and 690 nm (background) using a POLARstar Omega instrument (BMG LABTECH GmbH, Germany).

### *In vitro* scratch wound migration assay

PKF, IKF, NF and NS were seeded uniformly (5.0×10^4^ cells/well) into 6-well plates (with each well containing a single cover-slip) and grown for 240 hrs with conditioned media being replenished every ∼60 hrs. A wound scratch was made across the centre of each confluent cell culture using a sterile 200 µL pipette tip and any non-adherent cells were washed off with 1× PBS. Conditioned media was then reapplied to the cells according to the previous treatment regimen and the plates incubated for a further 30 hrs at 37°C/5% CO_2_. Cellular fixation was then achieved using 4% formaldehyde/PBS (Sigma-Aldrich, UK) applied for 30 min at RT, followed by permeabilisation with PBS/0.1% Triton X-100 solution (Sigma-Aldrich, UK). The cells were then treated with 1∶1000 diluted rhodamine phalloidin (Sigma-Aldrich, UK) and DAPI (Sigma-Aldrich, UK). Each well was photographed six times using objective inverted microscopy (Olympus, UK) and the number of cells that migrated into the scratched area counted (using a standardised scratch area for each image) as described previously [30].

### In Cell Western blotting (protein expression)

Following 240 hrs continuous conditioned-media treatments in 96-well plates, cells were fixed in 4% formaldehyde (Sigma-Aldrich, UK) for 20 min at room temperature. In-Cell Western blotting was carried out as described previously [1], briefly, After cells were fixed, the 96 well plates were washed three times with PBS (150 µL per well), permeabilized with PBS/0.1% TritonX-100 (150 µL per well, three times, 5 min each), and blocked in Odyssey blocking buffer (LI-COR, Cambridge, U.K.) (150 µL per well) for 2 h at room temperature. Primary antibodies used in this study included Collagen type I, Fibronectin, αSMA, TGF-β, CTGF and PAI-1 (**Table 2**). A mouse anti-α-tubulin primary antibody (Abcam, UK) was also used to simultaneously stain each well as a measure of determining housekeeping protein expression. All the primary antibodies incubation was carried out over night at 4°C. All rabbit primary antibodies were stained with IRDye800CW donkey anti-rabbit secondary antibody (Li-Cor, UK) and the mouse α-tubulin primary antibody was stained with anti-mouse IRDye680LT IgG. After staining with both, primary and secondary antibodies, the plates were imaged on an Odyssey infrared scanner (Li-Cor, UK) to measure protein expression readings at the respective wavelengths (800 nm for IRDye800CW and 700 nm for IRDye680LT) (**Table 3**). Expression of each protein marker was normalised to α-tubulin expression (800 nm/700 nm ratio) for each cell-media combination as described previously [3], [4], [30], [31]. Data were acquired by using Odyssey software, exported and analysed in Excel (Microsoft, Reading, U.K.)

**Table 2:** List of 1° Antibodies used in this study.

Antibody	Species Raised	Isotype	Dilution	Product Code	Source
**Collagen**	Rabbit polyclonal	IgG	1∶250	Ab292	Abcam
**Fibronectin**	Rabbit polyclonal	IgG	1∶500	Ab2413	Abcam
**α-SMA**	Mouse monoclonal	IgG2a	1∶500	A5691	Sigma-Aldrich
**PAI-1**	Rabbit polyclonal	IgG	1∶250	Ab66705	Abcam
**CTGF**	Rabbit polyclonal	IgG	1∶250	Ab6992	Abcam
**TGF-β2**	Rabbit polyclonal	IgG	1∶250	Ab66045	Abcam
**α-tubulin**	Mouse monoclonal	IgG1	1∶1000	Ab7291	Abcam

**Table 3:** List of 2° Antibodies used in this study.

Antibody	Raised Species	Isotype	Active against	Product Code	Source
**Donkey anti-mouse IRDye 800CW®**	Donkey	IgG	Mouse	926-32212	Li-Core
**Donkey anti-rabbit IRDye 800CW®**	Donkey	IgG	Rabbit	926-32213	Li-core
**Donkey anti-mouse IRDye 680CW®**	Donkey	IgG	Mouse	926-32222	Li-Core
**Donkey anti-rabbit IRDye 680CW®**	Donkey	IgG	Rabbit	926-32223	Li-Core

### RNA Extraction, cDNA synthesis and qRT-PCR (mRNA transcription)

RNA was extracted following the different treatment regimens (240 hrs) by washing cells in 1× PBS and treating with 0.5 mL Trizol (Invitrogen, UK) for 2 min at room temperature (RT). The resulting lysate was mixed with 0.2 mL chlorophorm for 2 min at RT and spun at 13,000 rpm for 15 min. The upper aqueous layer was collected and mixed with an equal volume of 70% (v/v) ethanol, from which total RNA was extracted with an RNeasy kit (Qiagen, UK) according to the manufacturer's instructions. DNase treatment was carried out using DNAfree kit (Ambion, UK). NanoDrop ND-1000 UV-visible spectrophotometer (Labtech International, UK) was used to estimate the total RNA concentration. RNA was normalised for all the cell samples to 250 ng for subsequent cDNA synthesis with qScript™ cDNA SuperMix (Quanta Biosciences, USA). Quantitative polymerase chain reactions were done in real-time using the LightCycler®480 II platform (Roche, UK). Each qRT-PCR reaction was carried out in a final volume of 10 µL, consisting of 4 µL diluted template cDNA, 5 µL Light Cycler 480 probes master mix (Roche Diagnostics, UK), 0.2 µM of forward and reverse primer (**Table 4**) (Sigma-Aldrich, UK), 1 µL probe from Universal Probe Library (Roche Diagnostics, UK) and 0.5 µL nuclease-free water (Ambion, UK). Each qRT-PCR reaction was done in triplicate with initiation at 95°C for 10 minutes to activate Hot Start *Taq* polymerase. 40 amplification cycles consisted of a 10-second denaturation step at 95°C and a 30-second annealing and elongation step at 60°C. Fluorescence intensity was recorded at the end of the annealing and elongation step in each cycle. A cooling step at 40°C for 30 seconds was carried out after the 40 cycles. Gene expression levels were normalized against an average of the internal reference gene, RPL-32, for each cell/media combination.

**Table 4:** Quantitative real time polymerase chain reaction (qRT-PCR) primers.

Gene/Primer	Gene ID	Sequence 5′ to 3′	Primer Position	Amplicon Size (bp)
**Collagen-I-L**	nm_000088.3	GGGATTCCCTGGACCTAAAG	1866–1885	63
**Collagen-I-R**	nm_000088.3	GGAACACCTCGCTCTCCA	1911–1928	
**Fibronectin-L**	nm_212482.1	GCCACTGGAGTCTTTACCACA	3498–3518	60
**Fibronectin-R**	nm_212482.1	CCTCGGTGTTGTAAGGTGGA	3539–3558	
**α-SMA-L**	nm_001613.1	CTGTTCCAGCCATCCTTCAT	834–853	70
**α-SMA-R**	nm_001613.1	TCATGATGCTGTTGTAGGTGGT	882–903	
**PAI-1-L**	nm_000602.1	AAACTCCCTAGTCTCCACCTGA	1473–1494	61
**PAI-1-R**	nm_000602.1	CCTTAAGGGAGTTGTGCTTCA	1513–1533	
**CTGF**	nm_001901.2	CTCCTGCAGGCTAGAGAAGC	884–903	93
**CTGF**	nm_001901.2	GATGCACTTTTTGCCCTTCTT	957–977	
**TGF β2-L**	nm_003238.1	CCAAAGGGTACAATGCCAAC	1194–1213	114
**TGF β2-R**	nm_003238.1	CAGATGCTTCTGGATTTATGGTATT	1283–1307	
**RPL32-L**	nm_000994.3	GAAGTTCCTGGTCCACAACG	319–338	76
**RPL32-R**	nm_000994.3	GAGCGATCTCGGCACAGTA	377–395	

### Statistical analysis of data sets

All statistical analyses were performed using the SPSS 13.0 software program (SPSS Inc., Chicago, IL, U.S.A.). All experiments were performed with at least 6 triplicates per condition. The mean of the triplicates was used for statistical analysis. The significance of the difference between the groups was analyzed statistically by two-way ANOVA with repeated measures. A Tukey's post hoc analysis was used in the case of significant effects. The difference between the means for all conditions was considered statistically significant at p<0.05.

## Results

### Peri-lesional keloid fibroblast conditioned media treatment induces increased spreading, proliferation and viability in normal scar and skin fibroblasts

**Figure 4:**
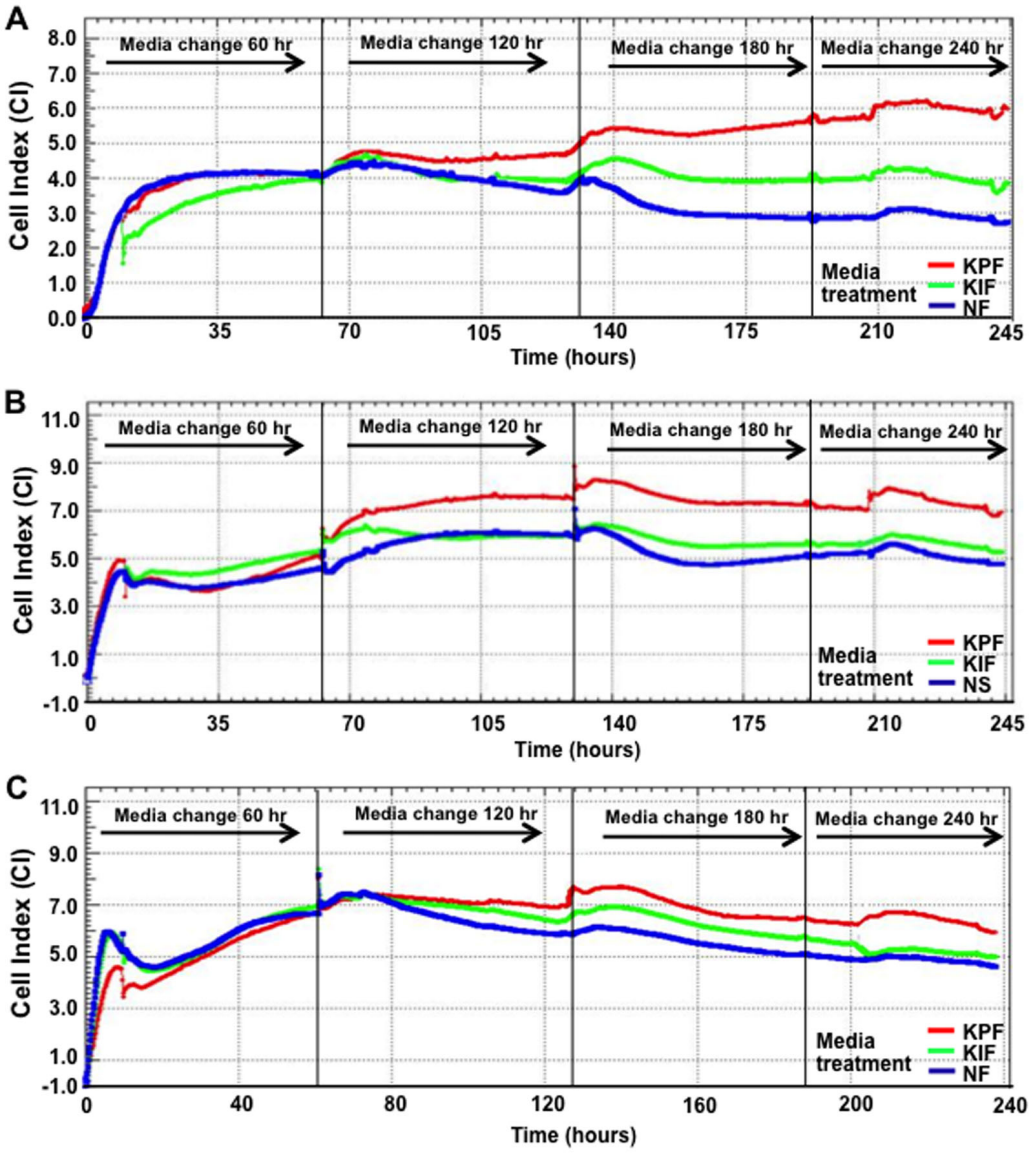
Real Time Cell Analysis (RTCA) over 240 hrs. **A.** Increased CI was observed at 60–240 hrs. PKF and IKF media treatment both elicited higher CI than NF media between 60–240 hrs. **B.** Similar trend for NS treated with PKF, IKF and NS control media was observed but with PKF media eliciting maximum CI at 120 hrs. **C.** Differences in CI were smaller for PKF with all media treatments, although overall CI values were greater at 60 hrs. CI = cell index, NF = normal dermal fibroblasts (n = 4), NS = Normal dermal scar fibroblasts (n = 4), PKF = peri-lesional keloid fibroblasts (n = 5), IKF = intra-lesional keloid fibroblasts (n = 5).

Cell index (CI) measured by the label free Real-Time Cell Analysis (RTCA) system is used as a guide to monitor cellular behaviour including spreading, proliferation, viability and morphology [29]. Dynamic responses in our panel of cells (to different conditioned media) were continuously monitored following 24 hrs cell synchronisation. NF cells treated with PKF conditioned media (**Figure 4A**) produced steady increases in CI from 60–240 hrs, indicating an enhanced NF cell spreading, proliferation and changes in cellular morphology (versus both IKF and control NF media). IKF media produced intermediate CI levels, below those for PKF but above NF media. Similar CI trends were observed for NS treated with PKF, IKF or control NS conditioned media (**Figure 4B**). The highest overall CI was from PKF (**Figure 4C**) followed by PKF, IKF and NF media treatments in which PKF media elicited consistently higher CI (above both IKF and NF treatments) for the duration of 240 hrs.

### Peri-lesional and intra-lesional keloid fibroblast conditioned media induce significantly elevated metabolic viability and proliferation in normal scar and skin fibroblasts

**Figure 5:**
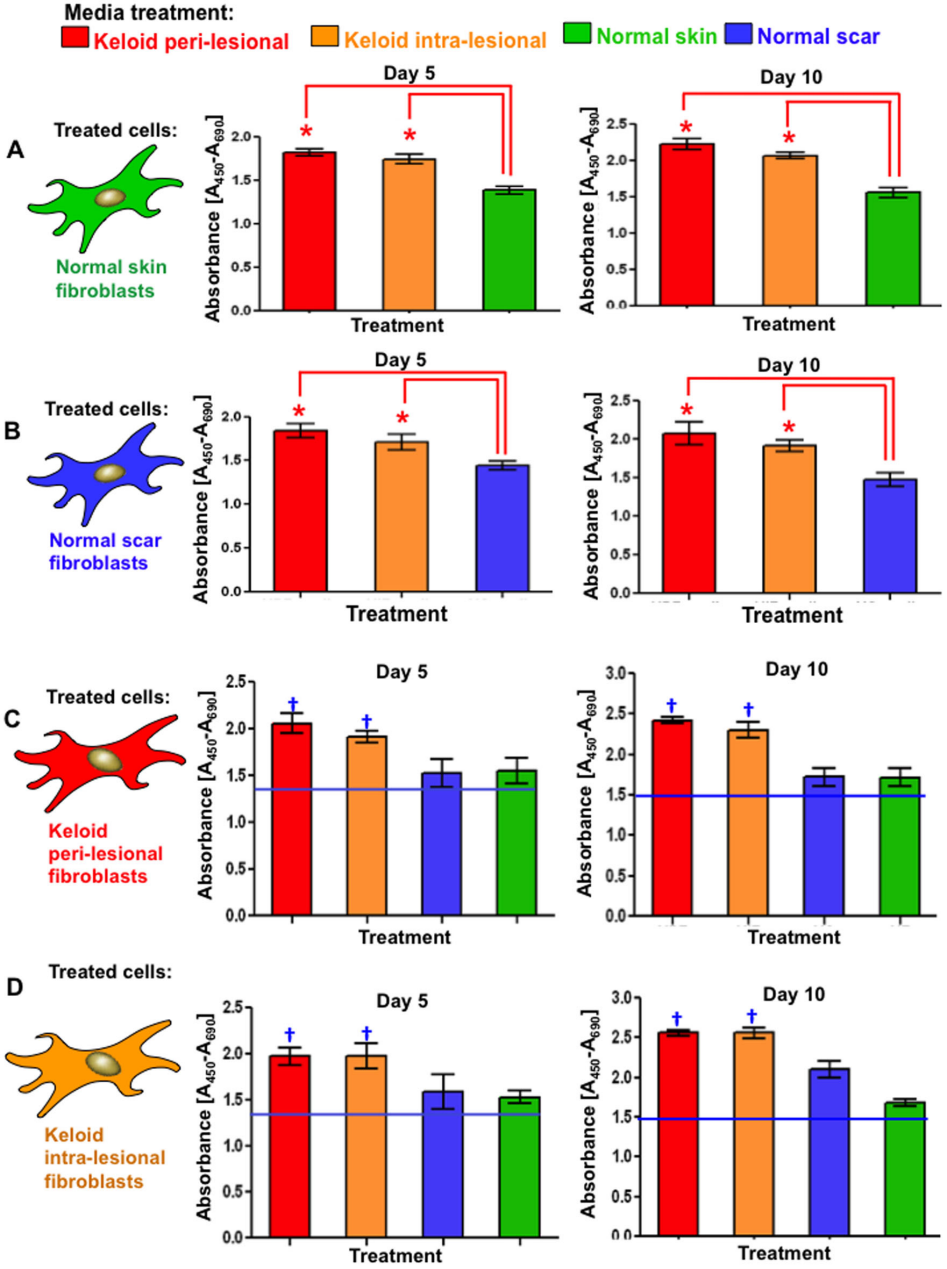
Proliferation after 120(day-5) and 240 hrs (day-10) of conditioned media treatment. **A.** Significantly increased (*p<0.03) proliferation and cellular viability was observed in both NF and NS treated with PKF or IKF media versus respective control media after 120 hrs. **B.** Similar trends were observed after 240 hrs although overall proliferation levels were higher than corresponding treatments at 120 hrs. **C.** Significantly higher proliferation was observed in PKF and IKF when treated with PKF or IKF media versus NF or NS media after 120 hrs. **D.** Similar trends were observed for PKF and IKF cells at 240 hrs with overall proliferation higher than corresponding treatments at 120 hrs. NF = normal dermal fibroblasts (n = 4), NS = Normal dermal scar fibroblasts (n = 4), PKF = peri-lesional keloid fibroblasts (n = 5), IKF = intra-lesional keloid fibroblasts (n = 5). Significantly increased (†p<0.02) proliferation and cellular viability was also observed in both PKF and IKF treated with PKF or IKF media versus respective NF and NS when treated with NF and NS media.

Conversion of the tetrazolium salt, WST-1, to a water-soluble formazan dye is dependent upon glycolytic bio-reduction of nicotinamide adenine dinucleotide (NADH) and was used as a direct measure of cell number/viability. Both PKF and IKF media treatments of NF elicited statistically significant (p<0.03) increased proliferation after 120 hrs compared to the NF media controls (**Figure 5A**). After 240 hrs significant increases were maintained (**Figure 5B**). After 120 hrs and 240 hrs, PKF media elicited consistently higher proliferation than IKF media. However, this difference was not statistically significant. Similar trends were observed in NS fibroblasts (**Figure 5A, 5B**). PKF and IKF showed statistically (p<0.03) elevated proliferation when treated with PKF or IKF media versus corresponding NF and NS control media (**Figure 5C, 5D**). Overall proliferation rates were greater, but not statistically significant, in PKF and IKF cells versus the corresponding NF and NS cell/media regimens.

### Increased migration in normal scar and skin fibroblasts treated with peri-lesional and intra-lesional keloid fibroblast conditioned media

**Figure 6:**
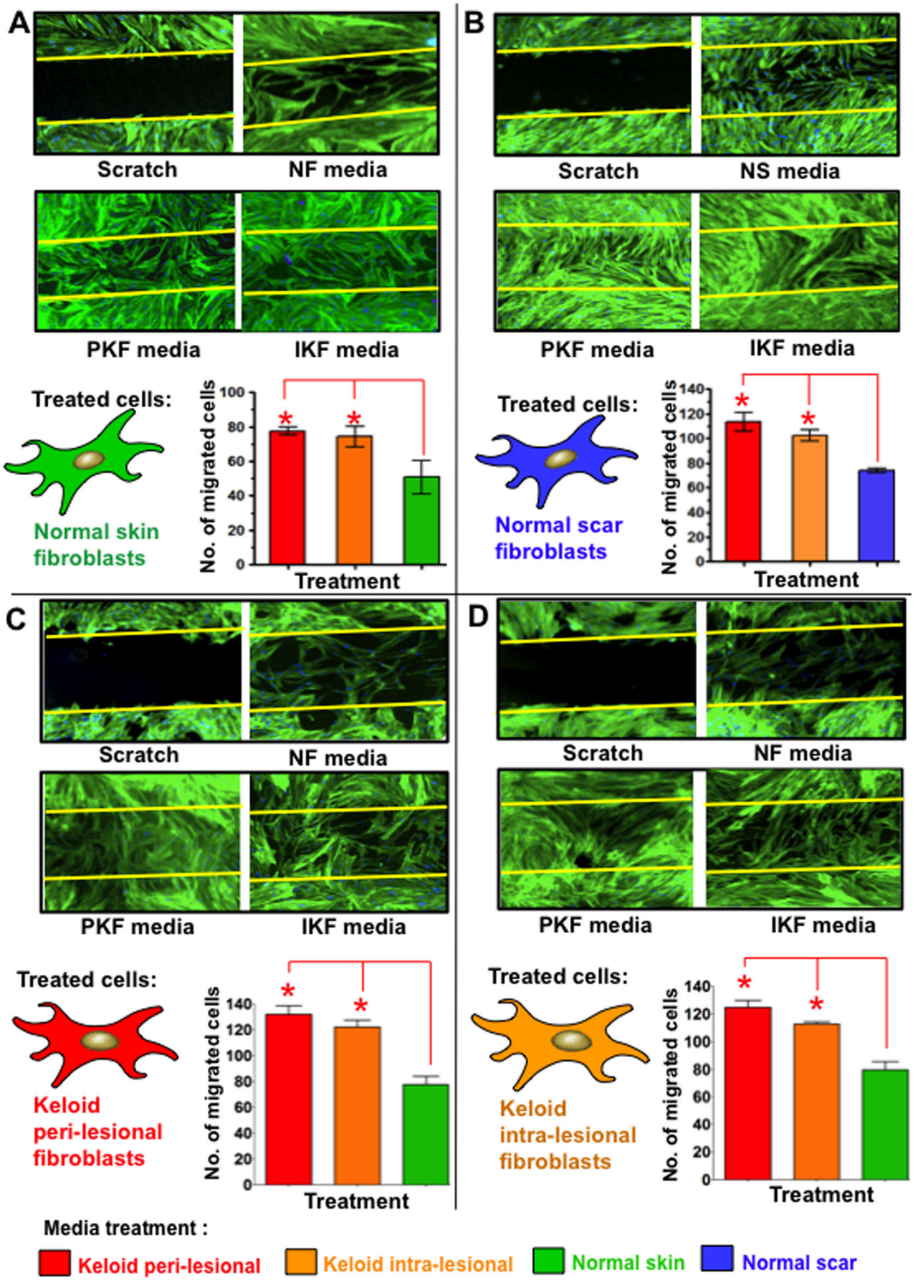
Cell migration in scratch wound invasion assay after 240 hrs of conditioned media treatment. **A.** Significantly increased (*p<0.03) cell migration occurred in NF over a 30 hrs period following 240 hrs treatment with PKF or IKF conditioned media versus NF control media. **B.** Significantly increased migration into a scratch wound was also observed in NS treated with PKF and IKF versus NS control media. **C.** PKF treated with NF control media formed a mesh-like network of cells in the scratch wound and significantly (p<0.01) increased migration following treatment with PKF and IKF conditioned media as compared to normal skin fibroblasts condition media. **D.** Significant (p<0.03) increased migration was also observed in IKF cells following treatments with PKF or IKF media versus NF control media. Blue = nuclei, Green = phalloidin stained intermediate filaments. NF = normal dermal fibroblasts (n = 4), NS = Normal dermal scar fibroblasts (n = 4), PKF = peri-lesional keloid fibroblasts (n = 5), IKF = intra-lesional keloid fibroblasts (n = 5).

**Figure 7:**
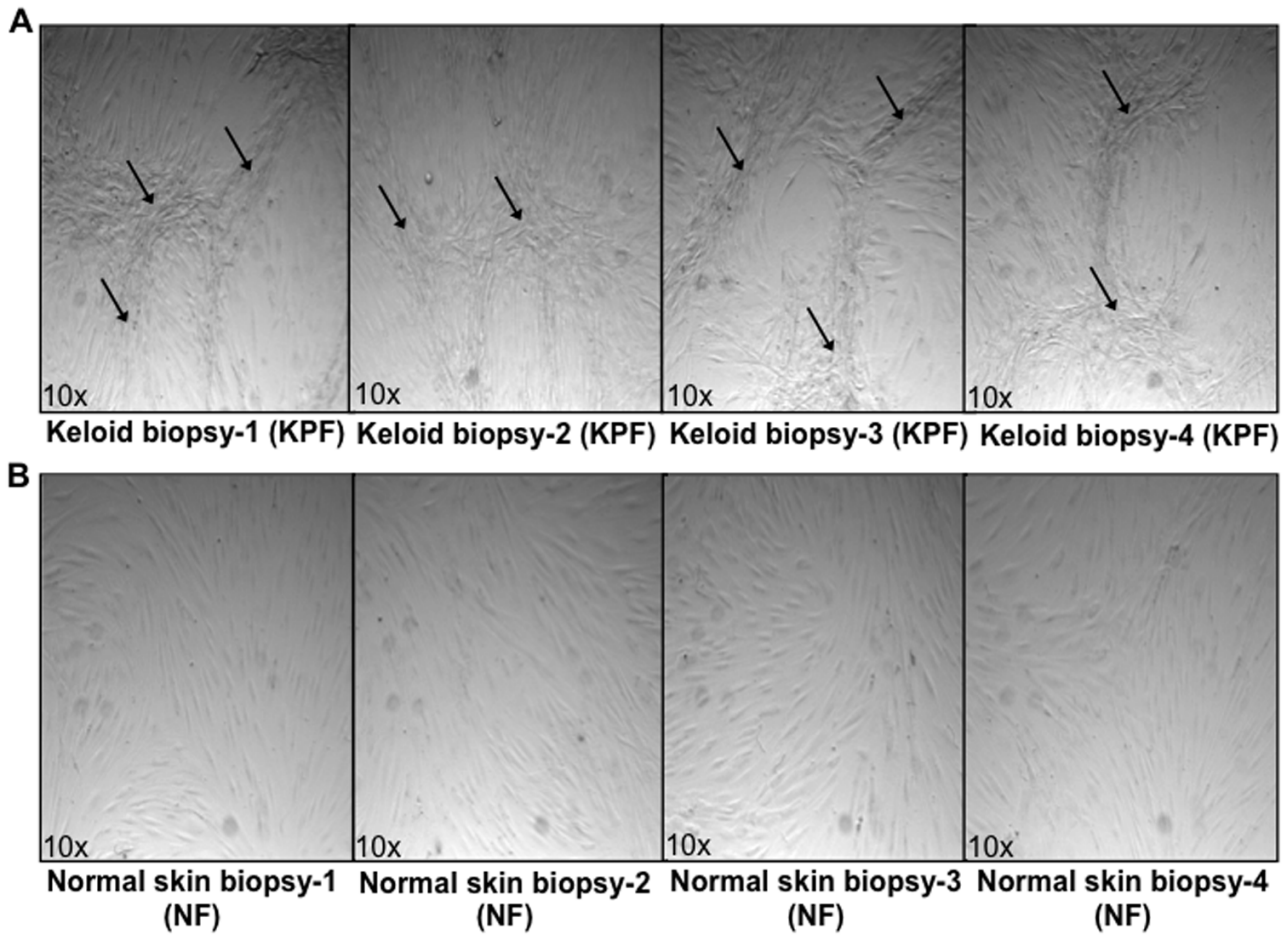
Cellular organisation in post-confluent *in vitro* primary cultures. **A.** Keloid primary fibroblasts derived from the peri-lesional margin of four keloid scar biopsies (PKF) passage ≤3. Arrows indicate whirl-like ridges and nodular aggregates formed when cells were grown to a post-confluent (>100%) state. **B.** Normal skin primary fibroblasts (NF) grown to a post-confluent (>100%) state did not show ridge structures or aggregates but grew in parallel layers.

Migration of NF (**Figure 6A**) and NS (**Figure 6B**) into a scratch wound inflicted across confluent cultures (following 240 of media treatments) was significantly elevated in PKF and IKF media-treated fibroblasts compared to NF or NS control media (p<0.04). Phalloidin staining of fibroblasts indicated cultures were confluent prior to scratching and both NF and NS cells orientated in parallel monolayers in contrast to both PKF and IKF cells, which formed whirl-like aggregates in a similar manner to the whirl-like nodular structures demonstrated in post-confluent cultures (**Figure 7**). PKF and IKF cells treated with PKF or IKF media both elicited strong migration into the scratch wound (**Figure 6C, 6D**).

### Peri-lesional and intra-lesional keloid fibroblast conditioned media induce elevated collagen I, FN, αSMA, CTFG, PAI-1 and TGFβ protein expression in normal scar and skin fibroblasts

**Figure 8:**
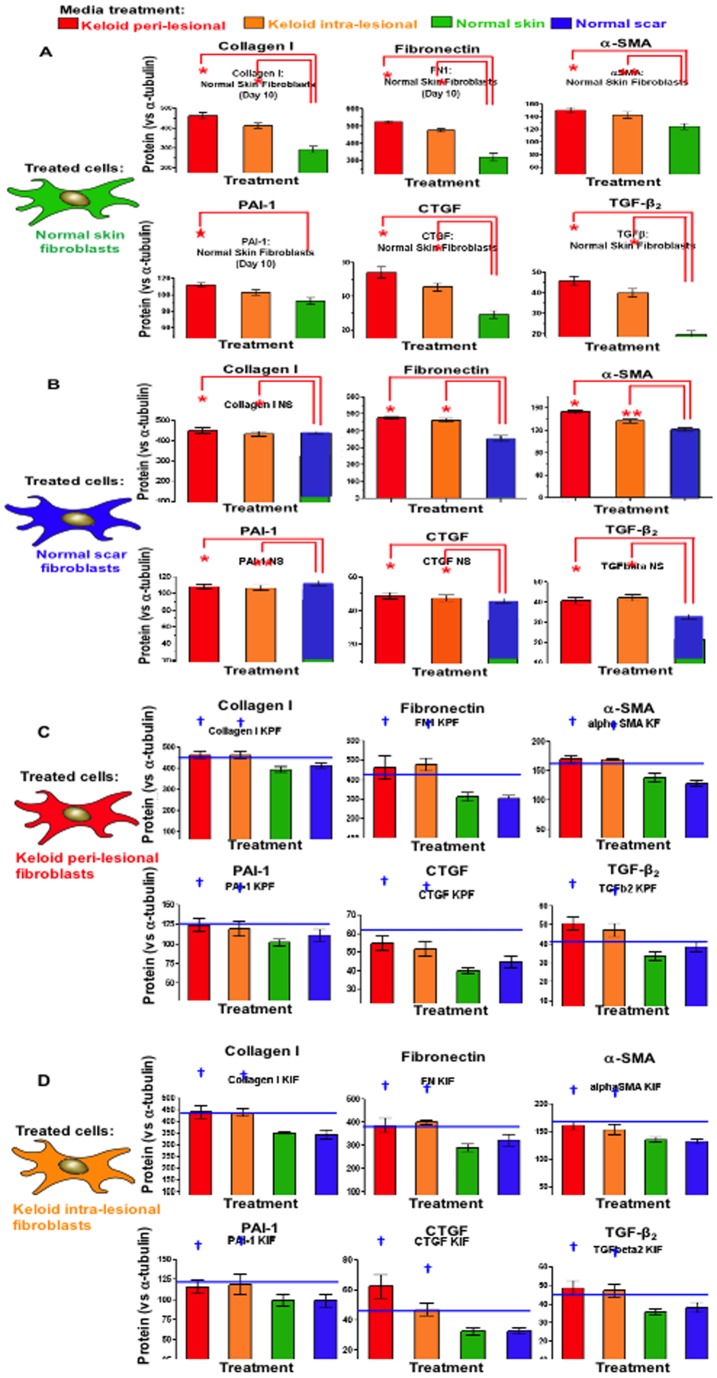
Protein expression after 240 hrs of replenishing conditioned media treatment. **A.** Significantly increased (*p<0.04) expression of collagen I, fibronectin, α-SMA, CTGF, PAI-1 and TGFβ-2 was observed in NF treated with PKF or IKF conditioned media versus NF control media after 240 hrs. Representative triplicates are shown below graphs (green) normalised against α-tubulin (red). **B.** Increased expression was observed for NS treated with PKF or IKF media versus NS control media after 240 hrs. **C.** PKF treated with PKF or IKF media elicited higher expression of all protein markers in comparison to both NF and NS media at 240 hrs. **D.** IKF treated with PKF or IKF media also increased expression compared to both NF and NS media at 240 hrs. NF = normal dermal fibroblasts (n = 4), NS = Normal dermal scar fibroblasts (n = 4), PKF = peri-lesional keloid fibroblasts (n = 5), IKF = intra-lesional keloid fibroblasts (n = 5). Significantly increased (†p<0.04) expression of collagen I, fibronectin, α-SMA, CTGF, PAI-1 and TGFβ was also observed in both PKF and IKF treated with PKF or IKF media versus respective NF and NS when treated with NF and NS media.

Protein expression for collagen I, FN, αSMA, CTGF, PAI-1 and TGFβ were significantly elevated (p<0.05) in NF following PKF or IKF media treatments versus NF control media after 240 hrs (**Figure 8A**). Despite collagen I, FN, αSMA CTGF and TGFβ expression consistently higher following PKF media treatments versus IKF media, no significant statistical difference existed between the two. Similar trends were observed in NS fibroblasts, with PKF and IKF media eliciting significant (p<0.04) increased expression in collagen I, fibronectin, αSMA, CTGF, PAI-1 and TGFβ versus NS control media (**Figure 8B**). Collagen I, FN, αSMA, CTGF, PAI-1 and TGFβ were all higher in PKF and IKF versus the corresponding treatments in NF or NS and both PKF and IKF media elicited increased expression versus NF/NS control media treatments (**Figure 8**).

### Peri-lesional and intra-lesional keloid fibroblast conditioned media induce elevated collagen I, fibronectin, α-SMA, CTGF, PAI-1 and TGFβ gene expression in normal scar and skin fibroblasts measured by quantitative real time (qRT)-PCR

**Figure 9:**
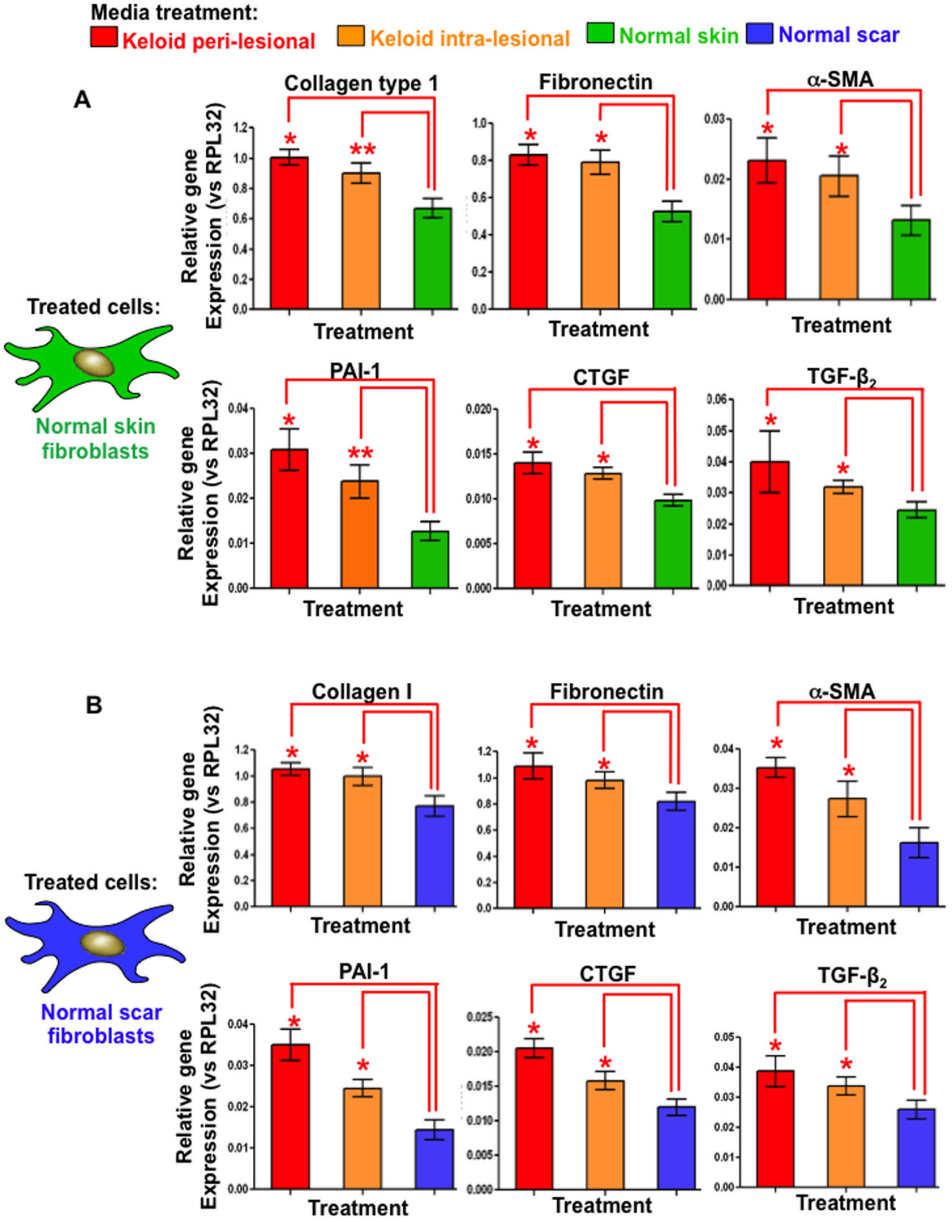
Gene expression measured by quantitative real time polymerase chain reaction (qRT-PCR) after 240 hrs of replenishing conditioned media treatment. **A.** Significantly increased (*p<0.03) expression of collagen I, fibronectin, α-SMA, CTGF, PAI-1 and TGFβ mRNA was observed in NF treated with PKF or IKF conditioned media (versus NF control conditioned media) after 240 hrs. All mRNA expression was normalised to the internal reference gene RPL-32. **B.** Increased gene expression was observed for NS treated with PKF or IKF conditioned media versus NS control media after 240 hrs. NF = normal dermal fibroblasts (n = 4), NS = normal dermal scar fibroblasts (n = 4), PKF = peri-lesional keloid fibroblasts (n = 5), IKF = intra-lesional keloid fibroblasts (n = 5).

mRNA expression for collagen I, FN, αSMA, CTGF, PAI-1 and TGFβ were significantly elevated (p<0.02) in NF following PKF or IKF media treatments versus NF control media after 240 hrs (**Figure 9A**). Similar trends were observed in NS fibroblasts, with PKF and IKF media eliciting significant increased expression in collagen I, fibronectin, αSMA, CTGF, PAI-1 and TGFβ versus NS control media (**Figure 9B**). Despite, the finding that collagen I, FN, αSMA CTGF, PAI-1 and TGFβ expression was consistently higher following PKF media treatments versus IKF media, no significant statistical difference was identified between the two.

## Discussion

Keloids scars are benign fibroproliferative dermal tumours of unknown origin that often occur following even the most minor form of trauma to the skin in genetically predisposed individuals [2]. The aetiopathogenesis of KD remains ill-defined, although a balance between keloid fibroblast (KF) proliferation and apoptosis is thought to influence both the accumulation of ECM and lesional invasion into the surrounding skin [32]. Region-specific KF growth-behaviour and ECM metabolism have previously been observed, wherein reticular dermal KF display reduced doubling times and increased cell-densities versus superficial and basal regions [32]. In our study, conditioned media from both marginal peri-lesional fibroblasts (PKF) and reticular dermal intra-lesional fibroblasts (IKF) elicited increased spreading and proliferation in both normal skin fibroblasts (NF) and normal scar fibroblasts (NS), as measured in real time (over 240 hrs) compared to their own control media (**Figure 4**). A corresponding, statistically significant (p<0.05) increase in cell proliferation and viability was elicited by PKF and IKF conditioned media at both 120 hrs and 240 hrs (**Figure 5A, 5B**) with concomitantly enhanced cell migration in an *in vitro* scratch wound assay (**Figure 6A, 6B**). Collectively, these results indicate PKF and IKF conditioned media can modify the cellular behaviour of normal non-disease fibroblasts through secreted paracrine mechanisms. Also, proliferation rates in PKF and IKF cells treated with control NS and NF media were above corresponding levels for NF and NS cells (**Figure 5C, 5D**). These levels were significantly increased upon PKF or IKF media treatment. Thus, secreted autocrine factors may well influence the shorter doubling times as previously reported for KF *in vitro* [21].

**Figure 10:**
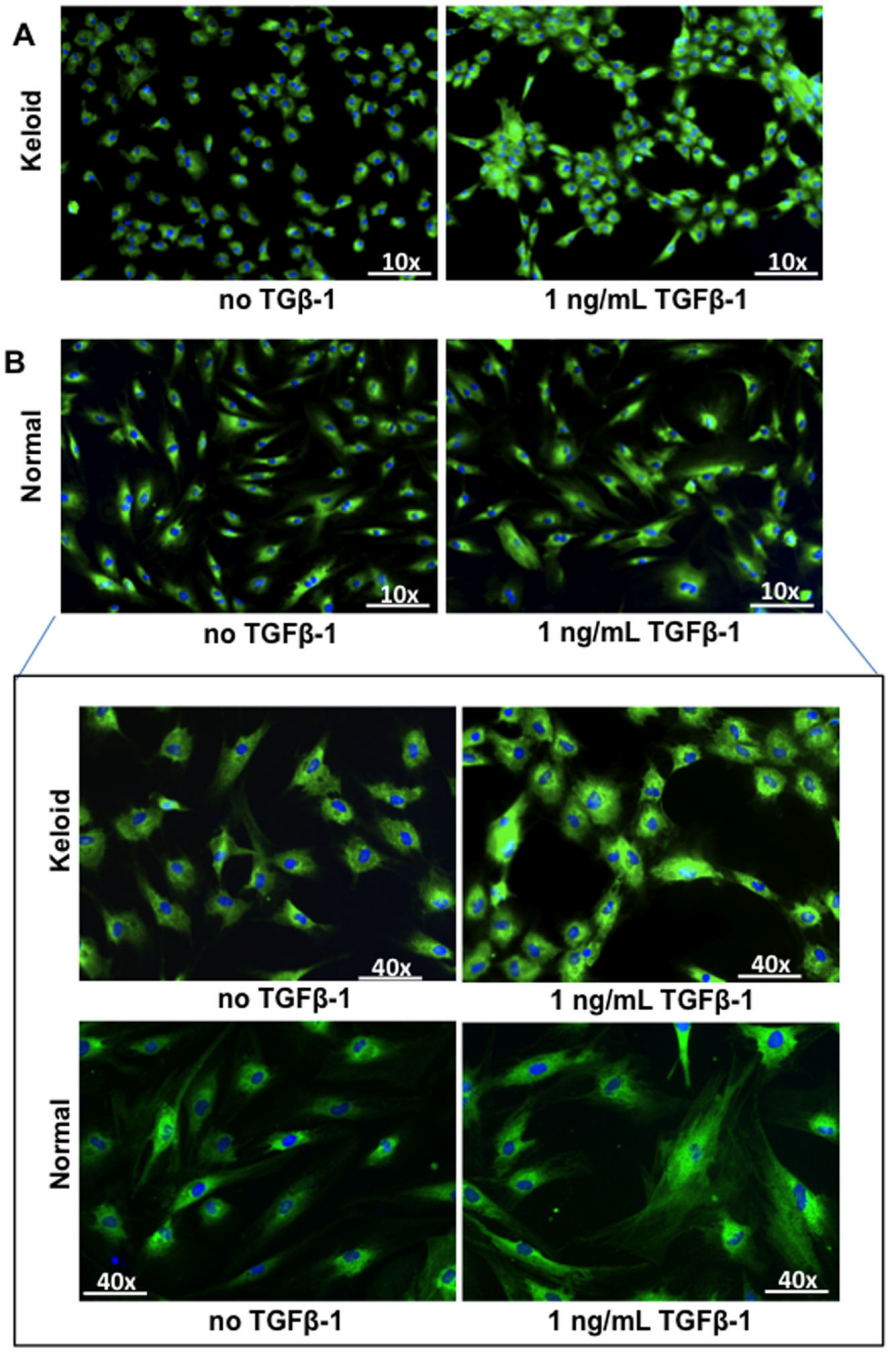
Effect of exogenous TGFβ-1 on α-smooth muscle actin-(SMA) expression and cellular organisation. **A.** Elevated α-SMA protein expression and cellular re-organisation into whirl-like structures were observed in PKF upon 24 hrs treatment with 1 ng/mL TGFβ-1. **B.** Elevated α-SMA expression and incorporation into microfilaments was observed in NF treated with 1 ng/mL TGFβ-1, although no cellular re-organisation was noted. Blue = nuclei, Green = α-SMA protein, NF = normal dermal fibroblasts (n = 4), PKF = peri-lesional keloid fibroblasts (n = 5).

We also observed PKF and IKF cells in long-term (post-confluent) *in vitro* culture, form a stratified-three dimensional structure, producing nodular aggregates mimicking the formation of keloid-like nodular tissue (**Figure 7**). These observations are consistent with previous reports [32], [33]. The whirl-like KF aggregates could also be induced within 24 hrs with exogenous 1 ng/mL TGFβ-1 (**Figure 10A**). These effects were not observed in NF (**Figure 10B**) indicating KF may have a dysregulated response to TGF-β signalling and/or respond through different TGFβ-mediated pathways [34], [35]. Indeed, KF cells treated with exogenous TGFβ-1 are known to stimulate significantly higher collagen I expression compared to NF [22], [23]. In our study, treatment of NF and NS with PKF or IKF media elicited higher TGFβ expression at both protein (**Figure 8A, 8B**) and mRNA (**Figure 9**) levels compared to control media. This observation is consistent with a statistically significant (p<0.03) increase in downstream protein markers associated with TGFβ-1-induction, including collagen I, fibronectin (FN), α-smooth muscle actin-(SMA), plasminogen activator inhibitor (PAI)-1, connective tissue growth factor (CTGF) and TGFβ (**Figure 8A, 8B**). Active *in situ* expression of collagen I and VI genes have previously been identified in lesional areas containing abundant KF (invading margin of the lesion) along with co-ordinate TGFβ mRNA and protein co-localisation [36]. In addition, it has been observed that PKF produce elevated levels of collagen I and III compared to both IKF and extra-lesional fibroblasts *in vitro* [1]. These results collectively suggest that IKF, and particularly marginal PKF, are likely to influence fibroblast behaviour through TGFβ-mediated signalling.

Interestingly, a synergistic effect of TGFβ and insulin-like growth factor (IGF)-1 co-stimulation is known to produce markedly higher KF expression of collagen I, FN and PAI-1 [23]. Affymetrix microarray studies also support the idea of several IGF-binding and IGF-related proteins being over-expressed in cultured KF [37], [38]. However, IGF-1 treatment alone has been shown to have no stimulatory effect upon KF, suggesting IGF-1 may enhance TGFβ-induced KD scar formation through TGFβ cross-talk. In our study, the increased expression of collagen I, FN and PAI-1 from NF and NS cells (treated with PKF or IKF media) may be a TGFβ-influenced effect although a synergistic role for PKF/IKF secreted IGF-1 would need to be experimentally confirmed. Paracrine signalling from KF may therefore involve a combination of secreted factors, acting in concert, rather than the effect of any one particular cytokine alone.

CTGF is a fibroblast-secreted, downstream effector of TGFβ signalling [39]. It is known to elicit fibroblast-specific mitogenesis, chemotaxis and ECM synthesis *in vitro* [40]. Gene array profiling has also indicated CTGF may be over-expressed in isolated KF treated with hydrocortisone [37]. In our study, the induction of significantly increased CTGF expression (p<0.05) in NF and NS, by both PKF and IKF conditioned media (**Figures 8A, 8B** **and** **9**) may account for the concomitant significant increase in cellular viability and proliferation versus normal conditioned media controls (**Figure 5B**). Observations of a stronger induction by PKF media above IKF media, although not statistically significant, complement reports of greater *in situ* concentrations of CTGF mRNA in KF from the scar periphery compared with a more diffuse distribution in reticular dermal KF [24]. Thus, elevated CTGF induction by PKF, and to a lesser extent IKF, may influence the localized behaviour of lesional fibroblasts and thus may facilitate KD invasion into the surrounding healthy skin.

FN is an integral glycoprotein component of the wound ECM and functions as a scaffold for collagens, which collectively contribute to the ECM network facilitating cell proliferation and migration. A relative steady-state increase in both intracellular and extracellular FN from KF *in vitro* was previously reported (versus NF) resulting from increased FN mRNA levels [41]. Also, exogenous rh-CTGF was shown to up-regulate both FN mRNA and protein (intracellular and secreted) in NF [42]. This induction was blocked by the pan-specific protein kinase-C (PKC) inhibitor, GF109203X, indicating CTGF-induced FN up-regulation is likely to be mediated through PKC secondary signalling [42]. Indeed, CTGF-mediated increases in collagen production have also been observed in NF [43]. We have shown treatment of both NF and NS, with PKF and IKF conditioned media, induced a statistically significant increase in the levels of both FN and collagen I expression at the protein (**Figure 8A, 8B**) and mRNA levels (**Figure 9A, 9B**). This increased expression may be a direct consequence of PKF/IKF secreted paracrine factors and/or a concomitant increase in CTGF secretion from NF and NS fibroblasts also induced by both PKF and IKF conditioned media over 240 hrs (**Figure 8A, 8B**). This effect may be exacerbated by the auto-inductive role of CTGF in stimulating its own up-regulation [42], [44]. In this scenario, secreted CTGF may increasingly stimulate higher endogenous CTGF expression, resulting in higher FN and collagen I accumulation in the ECM and, thus, maintenance of fibrosis.

TGFβ is known to induce Smad2/3 phosphorylation in KF promoting elevated PAI-1 mRNA expression mediated through several mitogen-activated protein kinase pathways including p38, ERK and JNK [45]. TGFβ has also been shown to stimulate increased PAI-1 in cultured NF *in vitro* [46]. In our study, PKF and IKF media both elicited increased PAI-1 in NF and NS at the protein (**Figure 8A, 8B**) and mRNA (**Figure 9**) levels, which may well result from corresponding increases in TGFβ (following the same treatment regimen) or through paracrine TGFβ from PKF/IKF. Both PKF and IKF cells produced higher endogenous PAI-1 than NF or NS when treated with keloid media, consistent with previous reports of elevated PAI-1 in KF [47]. Previous reports also indicate elevated PAI-1 is causally linked to elevated collagen synthesis in both NF and KF [48], supporting our observations of increased PAI-1 and collagen I. Aberrant PAI-1 in KD may thus contribute to fibrosis by enhanced suppression of the fibronolytic protease plasmin, mediated through inhibition of uPA, thus reducing the extent of fibrinolysis within a lesion.

Interleukin (IL)-6 is a cytokine whose expression is significantly higher in primary KF compared to NF [49]. Stimulation of NF with an IL-6 peptide is also known to produce a corresponding dose-dependent increase in Collagen type I α2 and FN1 mRNA [49]. Analysis of conditioned media from both NF and KF (non-culture) has previously indicated IL-6 to be the most abundant cytokine secreted by each cell type with the highest levels detected in KF [14]. These results suggest that IL-6 may be an important factor for eliciting PKF/IKF-induced paracrine effects as observed on both NF and NS. IL-8 was previously identified as the second most abundant cytokine secreted by KF with a significant increase versus NF. Additionally, two unique cytokines have been detected in conditioned media from KF which are absent from NF, namely hepatocyte growth factor (HGF) at day 5 and OncostatinM (OSM) at day 1 [14]. Further examination of this panel of cytokines indicated exogenous IL-6 and OSM elicited strong pY705 and pS727 Stat3 in both NF and KF, with OSM being the stronger inducer [14]. Stat3 is an oncogene and a latent transcription factor activated in cultured KF *in vitro* and thought to have an important role in keloid pathogenesis by promoting collagen expression, fibro-proliferation and cell migration [50], [51]. Inhibition of this Stat3 expression/phosphorylation in KF has shown reduced collagen production, impaired proliferation and delayed cell migration [50]. Thus, activation of Stat3 via chemokine, cytokine and/or growth factors secreted in PKF and IKF conditioned media may contribute to the fibrotic-like behaviour that we observe in NF and NS. Additionally, it may further enhance the activity of PKF and IKF fibroblasts *in vitro*, accounting for the generalised increase in proliferation, ECM and growth factor production when treated with their own respective conditioned media.

**Figure 11:**
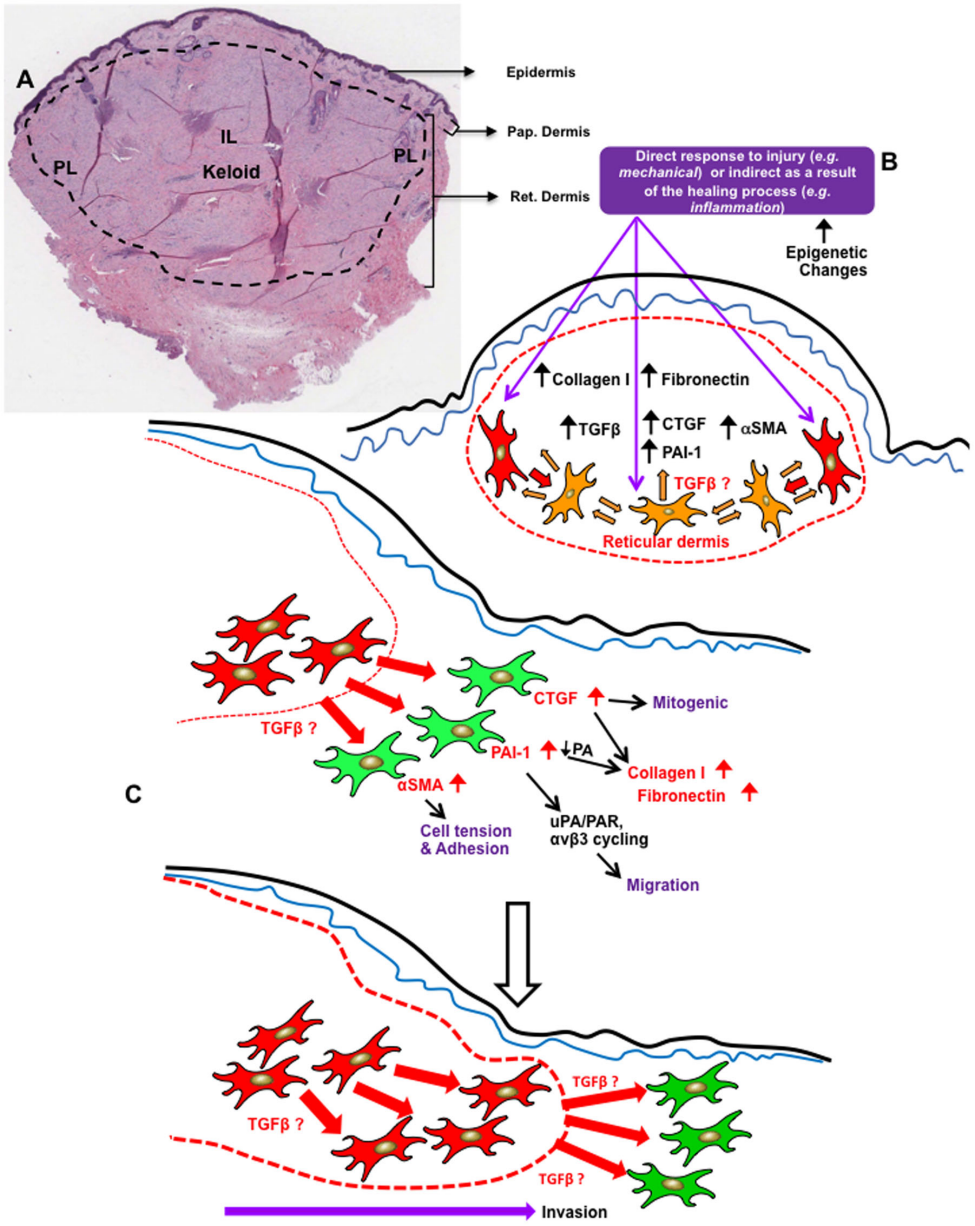
Proposed hypothetical mechanism of keloid recurrence. **A.** H&E showing cross section of Keloid lesion including adjacent normal skin. IL: intra-lesional; PL: peri-leisonal compartments; Pap. Dermis: papillary dermis; Ret. Dermis: Reticular dermis. **B.** Keloid fibroblasts may be changed, for example by mechanical injury itself or as a consequence of the healing process eg inflammation, and exhibit epigenetic differences to normal fibroblasts that allow paracrine signalling to occur. This may result in upregulated fibrotic markers that influence increased collagen and fibronectin deposition in a self-sustaining manner. **C.** At the margins of the lesion, keloid fibroblasts influence (paracrine signaling) normal skin primary fibroblasts to upregulate fibrotic markers such as CTGF, PAI-1, α-SMA. CTGF is known mitogenic for fibroblasts and PAI-1 is known to increase collagen deposition through inhibiting PA activity. CTGF may increase both collagen and fibronectin concomitantly. PAI-1 may also influence cell migration by stimulating PA receptor and β3 integrin cycling by endocytosis. α-SMA may also influence cell tension and adhesion. Collectively these changes may influence migration into the surrounding healthy skin leading to high recurrence of keloid lesion, post-surgery.

Our findings can be interpreted by postulating that keloid fibroblasts may be altered as a result of for example a mechanical injury or exhibiting epigenetic differences to normal fibroblasts that allow differential paracrine signalling to occur. This may subsequently result in up-regulation of fibrotic markers that may influence increased ECM deposition in a self-sustaining manner. At the margin of the keloid scar, these fibroblasts show strong paracrine signalling compared to normal skin fibroblasts which appear to result in upregulation of proteins such as CTGF, PAI-1, α-SMA. CTGF is a known mitogenic stimulant [52] for fibroblasts and PAI-1 is also known to increase collagen deposition through inhibiting PA activity [48]. In addition, CTGF may increase both collagen and fibronectin levels concomitantly. PAI-1 may also influence cell migration by stimulating PA receptor and beta3 integrin cycling by endocytosis [53]. Increased α-SMA may also influence cell tension and adhesion (our unpublished data). Collectively, these changes may influence migration into the surrounding healthy skin of keloid fibroblasts at the margin of the lesion, which may lead to the recurrence of keloid scarring (**Figure 11**).

In conclusion, primary NF and NS cells treated with PKF or IKF conditioned media exhibit enhanced expression of fibrosis-associated molecular markers and increased cellular activity as a result of keloid fibroblast-derived paracrine factors. KF autocrine/paracrine factors, in addition to genetic and epigenetic differences in KF as well as paracrine effects from inflammatory cells (and/or re-epithelialising keratinocytes) are all likely to exacerbate the abnormal KF secretory and/or responsive phenotype, resulting in an exuberant fibroblastic response. Further definition of the exact nature of KF autocrine and paracrine interactions may provide a progressively clearer understanding of the molecular mechanisms underlying aberrant KF activity and provide new targets for future therapeutic interventions.
